# Linear poly-ubiquitin remodels the proteome and influences hundreds of regulators in *Drosophila*

**DOI:** 10.1093/g3journal/jkae209

**Published:** 2024-09-26

**Authors:** Oluwademilade Nuga, Kristin Richardson, Nikhil C Patel, Xusheng Wang, Vishwajeeth Pagala, Anna Stephan, Junmin Peng, Fabio Demontis, Sokol V Todi

**Affiliations:** Department of Pharmacology, Wayne State University School of Medicine, 540 E. Canfield, Detroit, MI 48201, USA; Department of Pharmacology, Wayne State University School of Medicine, 540 E. Canfield, Detroit, MI 48201, USA; Department of Pharmacology, Wayne State University School of Medicine, 540 E. Canfield, Detroit, MI 48201, USA; Center for Proteomics and Metabolomics, St. Jude Children's Research Hospital, 262 Danny Thomas Place, Memphis, TN 38105, USA; Center for Proteomics and Metabolomics, St. Jude Children's Research Hospital, 262 Danny Thomas Place, Memphis, TN 38105, USA; Department of Developmental Neurobiology, St. Jude Children's Research Hospital, 262 Danny Thomas Place, Memphis, TN 38105, USA; Center for Proteomics and Metabolomics, St. Jude Children's Research Hospital, 262 Danny Thomas Place, Memphis, TN 38105, USA; Department of Developmental Neurobiology, St. Jude Children's Research Hospital, 262 Danny Thomas Place, Memphis, TN 38105, USA; Department of Structural Biology, St. Jude Children's Research Hospital, 262 Danny Thomas Place, Memphis, TN 38105, USA; Department of Developmental Neurobiology, St. Jude Children's Research Hospital, 262 Danny Thomas Place, Memphis, TN 38105, USA; Department of Pharmacology, Wayne State University School of Medicine, 540 E. Canfield, Detroit, MI 48201, USA; Department of Neurology, Wayne State University School of Medicine, 540 E. Canfield, Detroit, MI 48201, USA

**Keywords:** autophagy, catabolism, genetics, immune system, proteasome, proteomics

## Abstract

Ubiquitin controls many cellular processes via its posttranslational conjugation onto substrates. Its use is highly variable due to its ability to form poly-ubiquitin chains with various topologies. Among them, linear chains have emerged as important regulators of immune responses and protein degradation. Previous studies in *Drosophila melanogaster* found that expression of linear poly-ubiquitin that cannot be dismantled into single moieties leads to their ubiquitination and degradation or, alternatively, to their conjugation onto proteins. However, it remains largely unknown which proteins are sensitive to linear poly-ubiquitin. To address this question, here we expanded the toolkit to modulate linear chains and conducted ultra-deep coverage proteomics from flies that express noncleavable, linear chains comprising 2, 4, or 6 moieties. We found that these chains regulate shared and distinct cellular processes in *Drosophila* by impacting hundreds of proteins, such as the circadian factor Cryptochrome. Our results provide key insight into the proteome subsets and cellular pathways that are influenced by linear poly-ubiquitin chains with distinct lengths and suggest that the ubiquitin system is exceedingly pliable.

## Introduction

Myriad cellular processes and pathways in eukaryotic cells depend on the small modifier protein, ubiquitin (UB) (Komander and Rape 2012). UB is tethered chemically to other proteins posttranslationally. In essence, UB functions by changing the interaction landscape, protein folding, or both properties of its substrates. Because UB can be conjugated to other UB moieties, it can create various types of chain lengths, shapes, and sizes, including branched chains. A tremendous body of work conducted in vitro, in cellular systems, and in vivo has cataloged shared and distinct outcomes of the conjugation of different types of poly-UB to proteins and has also evidenced critical roles for unanchored, “free” ubiquitin chains that are linked head-to-toe, also known as linear UB chains (Pickart and Rose 1985; Hershko and Ciechanover 1998; Hicke *et al.* 2005; Zhou *et al.* 2005; Reyes-Turcu *et al.* 2008; Dikic *et al.* 2009; Kirkin *et al.* 2009; Komander *et al.* 2009; Laplantine *et al.* 2009; Xu, Duong *et al.* 2009; Inn *et al.* 2011; Braten *et al.* 2012; Clague *et al.* 2012; Komander and Rape 2012; Clague *et al.* 2013; Rivkin *et al.* 2013; Rajsbaum *et al.* 2014; Shimizu *et al.* 2015; Swatek and Komander 2016; Oh *et al.* 2018; Blount, Johnson *et al.* 2020; Tokunaga and Ikeda 2022; Gao *et al.* 2023; Sasaki and Iwai 2023; Sato *et al.* 2023).

**Fig. 1.**
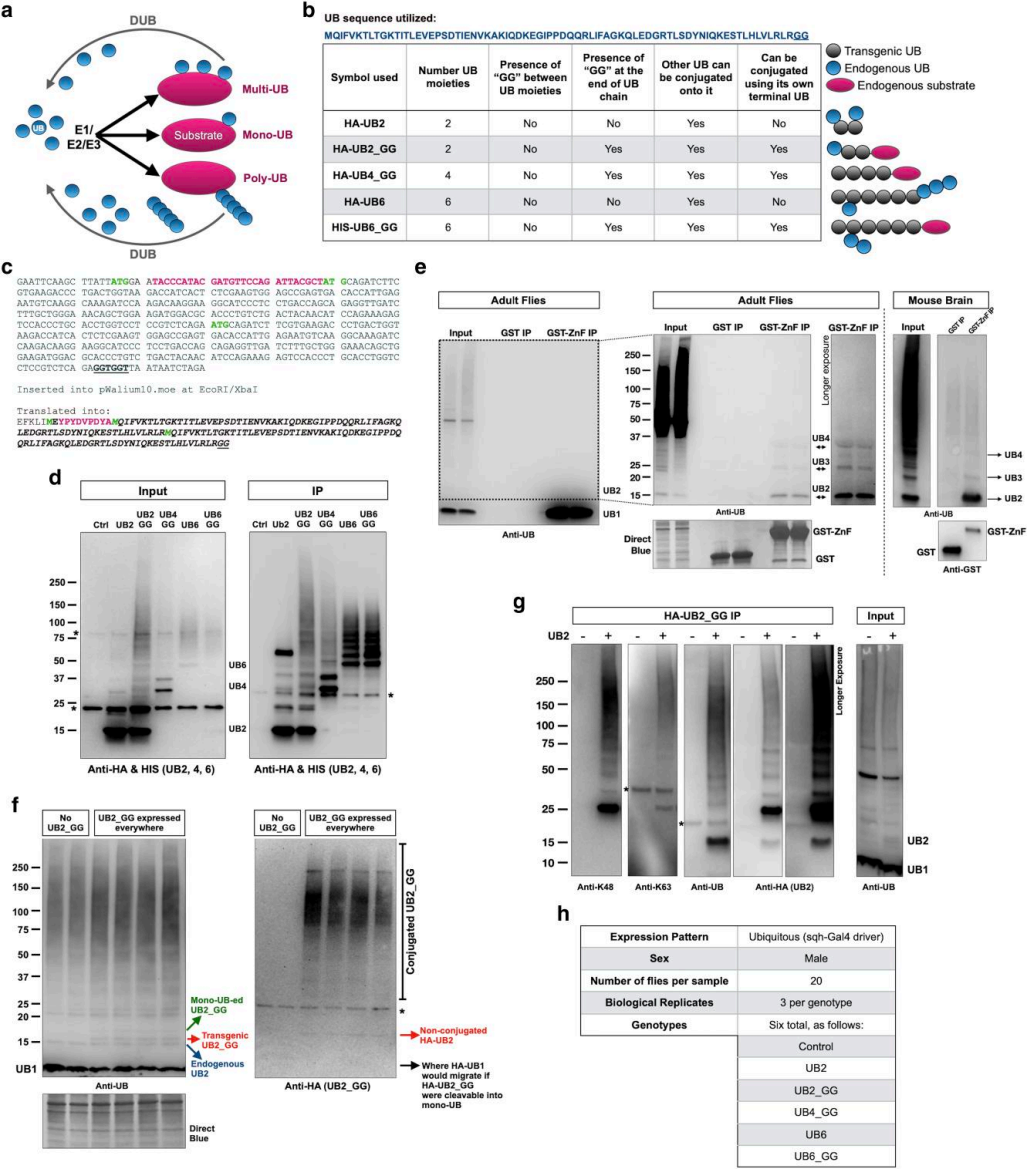
a) Graphical summary of the UB conjugation and deconjugation system. b) Tabulation of the different linear UB chains that were over-expressed in flies and their properties. Legend and graphics on the right portion of this panel indicate types of conjugation that may occur with these transgenic linear UB species. c) Nucleotide and amino acid sequence of the UB2_GG construct. The other lines generated for this project were designed similarly. The underlined portions show the terminal “GG” motif and the nucleotide sequence encoding it. The HA epitope tag is shown in magenta, and methionine residues corresponding to the first methionine of the construct and the first methionine residues of UB are in green. d) Western blots from the expression of the linear UB chains used in the study. The lysates were from male flies of the same crosses used for proteomics. Immunoprecipitation procedures are explained in the Materials and Methods section. “Ctrl” line was the sqh-Gal4 driver in trans with the host line used to generate the UBX transgenics. As noted in the main text, while UB2 and UB6 are not able to be conjugated onto other proteins, owing to the lack of their own terminal “GG” motifs, they can have endogenous UB conjugated onto them, leading to the signal above UB2 and UB6 in this panel. e) Isolation of unanchored UB using recombinant GST-tagged ZnF domain from USP5, processed as outlined in the Materials and Methods section. In Western blots from flies, the vast majority of unanchored UB was mono-UB. Cutting the membrane to remove this species (dotted box) and exposing it longer revealed other unanchored UB species. For blots from the mouse brain, mono-UB was cut off from the membrane to reveal the other chains. Mouse brain sample was leftover lysate from mice used in our prior publication (Todi *et al.* 2009). The input and IP lanes from the mouse brain lysate blots are from the same membrane and exposure, cropped and rearranged to ease visualization. f) Western blots from male flies expressing the noted UB chain. g) Immunoprecipitation and Western blots from flies expressing UB2_GG everywhere. h) Summary of the groups used for proteomics. In Western blots throughout this figure asterisks denote nonspecific bands. For full blots, please see Supplementary Fig. 1.

UB conjugation and de-conjugation are controlled by hundreds of enzymes. The coordinated action of a main E1 (UB-activating enzyme), of E2s (UB-conjugating enzymes) and E3s (UB ligases; Fig. 1a) ensures the chemical preparation and targeting of the 8.6 kDa UB protein to a specific amino acid on the substrate, most commonly a lysine (K) residue (Komander and Rape 2012). Errors in UB conjugation are resolved by deubiquitinases (DUBs), which are also tasked with UB recycling for maintaining a pool of free UB (Clague *et al.* 2012). The overall notion is that UB is conjugated in a stepwise manner onto a preceding UB to generate chains of different lengths and shapes. UB removal is conducted either one-by-one or en bloc, the latter then being dismantled to single UB proteins, ensuring a mono-UB pool for reutilization (Clague *et al.* 2012; Clague *et al.* 2013; Clague *et al.* 2019).

To expand the general understanding of the ways in which UB can function in vivo, we generated a series of transgenic *Drosophila melanogaster* UB chain-expressing lines that comprise 2, 4 (this study), or 6 UB (previously reported, Blount *et al.* 2018; Blount *et al.* 2019; Blount, Johnson, *et al.* 2020; Blount, Libohova, *et al.* 2020) in a head-to-toe arrangement. The fly lines are all isogenic to each other, with each transgene inserted into the attP40 site on chromosome 2 to ensure equal expression (Blount *et al.* 2018; Blount *et al.* 2019; Blount, Johnson, *et al.* 2020; Blount, Libohova, *et al.* 2020). These transgenic fly lines express distinct versions of linear poly-UB of different lengths that cannot be cleaved into mono-UB (Fig. 1b): one type lacks a terminal “GG” that would enable its conjugation onto another protein, whereas the other type contains a terminal “GG” motif and is able to be conjugated onto other proteins in vivo (Blount *et al.* 2018). Our prior studies with linear UB6 indicated that expression of noncleavable, linear poly-UB is not detrimental to *Drosophila*, regardless of the expression pattern; that these chains are degraded by the proteasome; that chains with a terminal “GG” can be conjugated onto other proteins; and that chains without a terminal “GG” are decorated by numerous types of linkages comprising endogenous UB (Blount *et al.* 2018; Blount *et al.* 2019; Blount, Johnson, *et al.* 2020; Blount, Libohova, *et al.* 2020). Collectively, the expression of these exogenous chains suggests a highly flexible and adaptable UB system, able to handle linear UB chains that cannot be removed via the action of DUBs (Blount, Johnson, *et al.* 2020). However, a key outstanding question is whether there are specific sets of proteins that are particularly sensitive to modulation by linear UB chains.

Here, we addressed this gap in knowledge by identifying the cellular processes and pathways that are impacted by the expression of distinct linear poly-UB that can or cannot be conjugated onto protein substrates. We hypothesized that some of these UB chains could be utilized to enhance the degradation of specific proteins, and that UB chains that cannot be conjugated onto other proteins could also regulate cellular processes independently from protein degradation. Through the utilization of quantitative, unbiased proteomics (Peng *et al.* 2003) of male flies that expressed UB2, UB4, or UB6 chains ubiquitously, we found that linear UB chains distinctly influence cellular processes and molecular functions. Collectively, our results expand the appreciation of the organism's flexible use of UB and identify proteins that are particularly sensitive to modulation by linear poly-UB.

## Materials and methods

### Antibodies

Anti-HA (rabbit monoclonal C29F4, 1:500–1,000; Cell Signaling Technology); anti-HIS (rabbit monoclonal, D3I1O; 1:500, Cell Signaling Technology); anti-ubiquitin (mouse monoclonal A5, 1:1,000, Santa Cruz Biotech); anti-K48 ubiquitin (rabbit monoclonal D9D5, 1:500–1,000; Cell Signaling Technology); anti-K63 ubiquitin (rabbit monoclonal DA7011, 1:500–1,000; Cell Signaling Technology). HRP-conjugated secondary antibodies were used at 1:5,000 and were from Jackson ImmunoResearch.

### *Drosophila*-related procedures

Flies were mated, reared, and maintained in diurnally controlled environments at 25˚C. We used fly media consisting of 2% agar; 10% yeast; 10% sugar. The transgenic UB6 and UB6_GG lines were previously reported (Blount *et al.* 2018; Blount *et al.* 2019; Blount, Libohova, *et al.* 2020). The UB2, UB2_GG, and UB4_GG lines were generated similarly, by sub-cloning the intended linear UB construct into pWalium10.moe (DNA Resource Core at Harvard Medical School). The precise nucleotide sequence used is shown for UB2_GG in Fig. 1c. The UB4_GG nucleotide sequence used was a near-duplication of the UB2_GG: 5′—gaattcaagc ttattatgga atacccatac gatgttccag attacgctat gcagatcttc gtgaagaccc tgactggtaa gaccatcact ctcgaagtgg agccgagtga caccattgag aatgtcaagg caaagatcca agacaaggaa ggcatccctc ctgaccagca gaggttgatc tttgctggga aacagctgga agatggacgc accctgtctg actacaacat ccagaaagag tccaccctgc acctggtcctc cgtctcagaa tgcagatcttc gtgaagaccc tgactggtaa gaccatcact ctcgaagtgg agccgagtga caccattgag aatgtcaagg caaagatcca agacaaggaa ggcatccctc ctgaccagca gaggttgatc tttgctggga aacagctgga agatggacgc accctgtctg actacaacat ccagaaagag tccaccctgc acctggtcct ccgtctcaga atggaatacc catacgatgt tccagattac gctatgcaga tcttcgtgaa gaccctgact ggtaagacca tcactctcga agtggagccg agtgacacca ttgagaatgt caaggcaaag atccaagaca aggaaggcat ccctcctgac cagcagaggt tgatctttgc tgggaaacag ctggaagatg gacgcaccct gtctgactac aacatccaga aagagtccac cctgcacctg gtcctccgtc tcagaatgca gatcttcgtg aagaccctga ctggtaagac catcactctc gaagtggagc cgagtgacac cattgagaat gtcaaggcaa agatccaaga caaggaaggc atccctcctg accagcagag gttgatctttg ctgggaaac agctggaaga tggacgcacc ctgtctgact acaacatcca gaaagagtcc accctgcacc tggtcctccg tctcagaggt ggttaataat ctaga—3′.

For UB2 lacking a terminal “GG”, the final construct lacked the underlined portion of the sequence in Fig. 1c. Transgenic flies were generated through PhiC-31-dependent integration of pWalium10.moe into attP40 site on chromosome 2 of the fly. Upon transformation and confirmation of the insertion and its encompassing sequence via PCR and genomic sequencing, all lines were transferred onto the w^1118^ background and were backcrossed for multiple generations before being utilized for downstream crosses. The sqh-Gal4 line was a gift of Dr. Daniel Kiehart (Duke University) (Karess *et al.* 1991; Franke *et al.* 2005; Todi *et al.* 2005).

### Western blotting

Five male flies per group were homogenized in hot SDS lysis buffer (50 mM Tris pH 6.8, 2% SDS, 10% glycerol, 100 mM dithiothreitol (DTT)), sonicated, boiled for 10 minutes, and then centrifuged for 10 minutes at 13,300 rpm at room temperature. Samples were electrophoresed using Tris/Glycine gels (Bio-Rad). Western blot imaging was conducted using ChemiDoc (Bio- Rad). For loading controls, we used total protein staining by saturating PVDF membranes for 10 min in 0.008% Direct Blue 71 (Sigma-Aldrich) dissolved in 40% ethanol and 10% acetic acid, rinsed with a solution of 40% ethanol/10% acetic acid, then ultra-pure water, and then air dried and imaged. Supplementary Fig. 1 shows un-cropped blots.

### Immunoprecipitation

For panels Fig. 1d and g, 10 adult males were homogenized in 500 µL of NETN lysis buffer (50 mM Tris, pH 7.5, 150 mM NaCl, 0.5% Nonidet P-40) supplemented with protease inhibitors (PI without EDTA; Sigma-Aldrich). The homogenates were then centrifuged for 10 minutes at 10,000×g at 4˚C. The supernatant was subsequently combined with bead-bound anti-HA antibody for all species, except UB6_GG, which was incubated with Ni-NTA beads (Fig. 1b; Sigma-Aldrich) and tumbled at 4˚C for 4 h. Beads were rinsed 5 times each with lysis buffer. Bead-bound complexes were eluted through Laemmli buffer (Bio-Rad) and heating at 95˚C for 5 minutes. The supernatant was supplemented with 6% SDS to a final concentration of 1.5% SDS and loaded for Western blotting. For immunoprecipitation data shown in Fig. 1e, the recombinant protein (GST alone or GST-ZnF) was immobilized on glutathione-sepharose beads using protocols we have described in the past (Ristic *et al.* 2016), and then was supplemented with *Drosophila* lysates (10 whole flies per sample) or whole mouse brain lysates homogenized in NETN + PI at a concentration of 1 mg/mL. Incubation was conducted at 4˚C, tumbling for 4 h. Beads were then rinsed 5 times with the lysis buffer and complexes were eluted through Laemmli buffer (Bio-Rad) and heating at 95˚C for 5 min.

### Protein sample preparation, protein digestion, and peptide isobaric labeling by tandem mass tags

For each TMT *Drosophila* sample, 20 male flies at 10 days of age were collected and homogenized in 8 M urea lysis buffer (50 mM HEPES, pH 8.5, 8 M urea) (Hunt *et al.* 2019; Jiao *et al.* 2021; Hunt *et al.* 2023; Hunt *et al.* 2024). We utilized 10-day-old flies because these are flies with optimal Gal4-mediated transgene expression and are unencumbered by the physiological changes that occur immediately posteclosion. After homogenization with zirconium beads in a NextAdvance bullet blender, 0.5% sodium deoxycholate was added to the tissue homogenates, which were then briefly pelleted to remove cuticle debris. The protein concentration of the lysates was determined by Coomassie-stained short gels using bovine serum albumin (BSA) as standard, as previously described (Xu, Duong *et al.* 2009). For TMT mass spectrometry, 100 µg of each sample was digested with LysC (Wako) at an enzyme-to-substrate ratio of 1:100 (w/w) for 2 h in the presence of 1 mM DTT. Following this, the samples were diluted to a final 2 M Urea concentration with 50 mM HEPES (pH 8.5) and further digested with trypsin (Promega) at an enzyme-to-substrate ratio of 1:50 (w/w) for at least 3 h. The peptides were reduced by adding 1 mM DTT for 30 min at room temperature (RT) followed by alkylation with 10 mM iodoacetamide (IAA) for 30 min in the dark at RT. The unreacted IAA was quenched with 30 mM DTT for 30 min. Finally, the digestion was terminated and acidified by adding trifluoroacetic acid (TFA) to 1%, desalted using C18 cartridges (Harvard Apparatus), and dried by speed vac. The purified peptides were resuspended in 50 mM HEPES (pH 8.5) and labeled with 18-plex Tandem Mass Tag (TMTpro) reagents (ThermoScientific) following the manufacturer's recommendations and our optimized protocol (Wang *et al.* 2020).

### Two-dimensional HPLC and mass spectrometry

The TMT-labeled samples were mixed equally, desalted, and fractionated on an offline HPLC (Agilent 1220) by using basic pH reverse-phase liquid chromatography (pH 8.0, XBridge C18 column, 4.6 mm × 25 cm, 3.5 μm particle size, Waters). The fractions were dried and resuspended in 5% formic acid and analyzed by acidic pH reverse phase LC-MS/MS analysis. The peptide samples were loaded on a nanoscale capillary reverse phase C18 column (Thermo PepMap RSLC column, 75 um ID × 15 cm, 2 μm C18 resin) by an HPLC system (Thermo Ultimate 3000) and eluted by a 100-min gradient. The eluted peptides were ionized by electrospray ionization and detected by an inline Q-Exactive HF mass spectrometer (ThermoScientific). The mass spectrometer was operated in data-dependent mode with a survey scan in Orbitrap (60,000 resolution, 1 × 10^6^ AGC target and 50 ms maximal ion time) and MS/MS high-resolution scans (60,000 resolution, 1 × 10^5^ AGC target, 110 ms maximal ion time, 32 HCD normalized collision energy, 1 *m*/*z* isolation window, and 10 s dynamic exclusion).

### MS data analyses

The MS/MS raw files were processed by the tag-based hybrid search engine, JUMP (Wang *et al.* 2014). The raw data were searched against the UniProt *Drosophila* databases concatenated with a reversed decoy database for evaluating false discovery rates. Searches were performed by using a 25-ppm mass tolerance for both precursor and product ions, fully tryptic restriction with two maximal missed cleavages, three maximal modification sites, and the assignment of *a*, *b*, and *y* ions. TMT tags on Lys and N-termini (+304.20715 Da) were used for static modifications and Met oxidation (+15.99492 Da) was considered as a dynamic modification. Matched MS/MS spectra were filtered by mass accuracy and matching scores to reduce protein false discovery rate to ∼1%. Proteins were quantified by summing reporter ion intensities across all matched PSMs using the JUMP software suite (Pagala *et al.* 2015). The mass spectrometry proteomics data have been deposited to the ProteomeXchange Consortium via the PRIDE partner repository with the dataset identifier PXD052020.

### Supervised and over-representation analysis

Raw values from MS data analyses were used to run a PCA using the “prcomp” function in R software. Log2FC values of the UBX group compared to control were used to plot a heatmap (ComplexHeatmaps in R) (Gu *et al.* 2016a; Gu *et al.* 2016b; Gu *et al.* 2016c) using protein IDs curated from flybase.org (Ozturk-Colak *et al.* 2024) and annotated to relevant pathways addressed in the text. Differentially-abundant proteins were identified as proteins that were significantly different from control (*P* < 0.05) with log2FC ≥ 0.5. GeneOverlap package (https://rdrr.io/bioc/GeneOverlap/2024) was used to visualize protein ID overlaps and to calculate significance using a genome size background of 8,000 genes. Differentially abundant genes were used to determine pathway enrichment by employing GeneOntology (Ashburner *et al.* 2000; Carbon *et al.* 2009; Thomas *et al.* 2022; Aleksander *et al.* 2023), Kyoto Encyclopedia of Genes and Genomes (KEGG) (Kanehisa and Goto 2000; Kanehisa 2019; Kanehisa *et al.* 2023), and the clusterprofiler (Yu *et al.* 2012; Wu *et al.* 2021) packages in R.

## Results

### Expression of different linear UB chains in *Drosophila*

We previously reported the generation of transgenic fly lines containing UB6 chains that cannot be dismantled due to the absence of “GG” motifs between constituent moieties (Blount *et al.* 2018; Blount *et al.* 2019; Blount, Libohova, *et al.* 2020). These chains (Fig. 1b; 1c details UB2; all the other lines were generated similarly) were designed to lack (or contain) a final “GG”, meaning that they could not (or could) themselves become conjugated onto endogenous proteins in the fly through this terminal motif. Here, we expanded the toolkit to examine the function of linear UB chains by generating three additional transgenic fly models containing UB2 linear chains without or with the terminal “GG” and UB4 with a terminal “GG”. All of these lines are isogenic and were site-integrated (with the PhiC31 system (Groth *et al.* 2004)) into attP40 on *Drosophila* chromosome 2 to ensure equal expression. Each line is expressed through the binary Gal4-UAS system (Brand and Perrimon 1993) and the sqh-Gal4 driver, which enables transgene expression in all fly tissues, throughout development and in adulthood (Karess *et al.* 1991; Franke *et al.* 2005; Todi *et al.* 2005; Tsou *et al.* 2012). All analyses were conducted with male flies that were 10 days old. Figure 1d shows the expression of the various UB transgenes in these flies. We note that UB2 and UB6, which lack a terminal “GG” motif and therefore cannot be conjugated onto other proteins, can themselves be modified by endogenous UB, leading to bands and smears above their respective, primary bands. We detailed this outcome previously for UB6 (Blount *et al.* 2018).

We decided to generate transgenes that express UB2, UB4, and UB6 chains for the following reasons. UB4 is generally accepted as the minimal length required to target a substrate for proteasomal degradation effectively (Thrower *et al.* 2000). Based on biochemical work that we conducted with whole fly tissue or mouse brain lysates, we noticed that among unanchored chains isolated using the Zinc finger domain of USP5, which binds unanchored UB (Wilkinson *et al.* 1995; Reyes-Turcu *et al.* 2008), UB2 was the most prominent species (Fig. 1e; please note that UB1 was vastly more abundant than unanchored UB2 in this assay). We also reasoned that it would be informative to examine the effect of the next-largest building block of UB, after UB1, in the overall proteome. As shown in Fig. 1f and g, UB2_GG is tethered with other proteins in the fly and is used to generate mixed-linkage chains, indicating its incorporation into the UB pool in vivo. Lastly, we thought it reasonable to also test UB6 in this study, both as a logical longer UB chain and because we had tested its impact on the fly before (Blount *et al.* 2018; Blount *et al.* 2019; Blount, Libohova, *et al.* 2020). With these tools on hand, we proceeded to conduct unbiased, quantitative proteomic analyses.

### TMT mass spectrometry of fruit flies expressing noncleavable linear UB chains

We conducted 18-plex tandem mass tags (TMT) mass spectrometry from flies expressing the above-mentioned linear UB chains ubiquitously during development and in adulthood. We used the same driver that we have used in other studies with these chains, sqh-Gal4. The groups that we used for examination were: UB2, UB2_GG, UB4_GG, UB6, and UB6_GG (Fig. 1h). The control group with no UB chain expression consisted of flies with the sqh-Gal4 driver on the same genetic background as the linear UB transgenics.

**Fig. 2.**
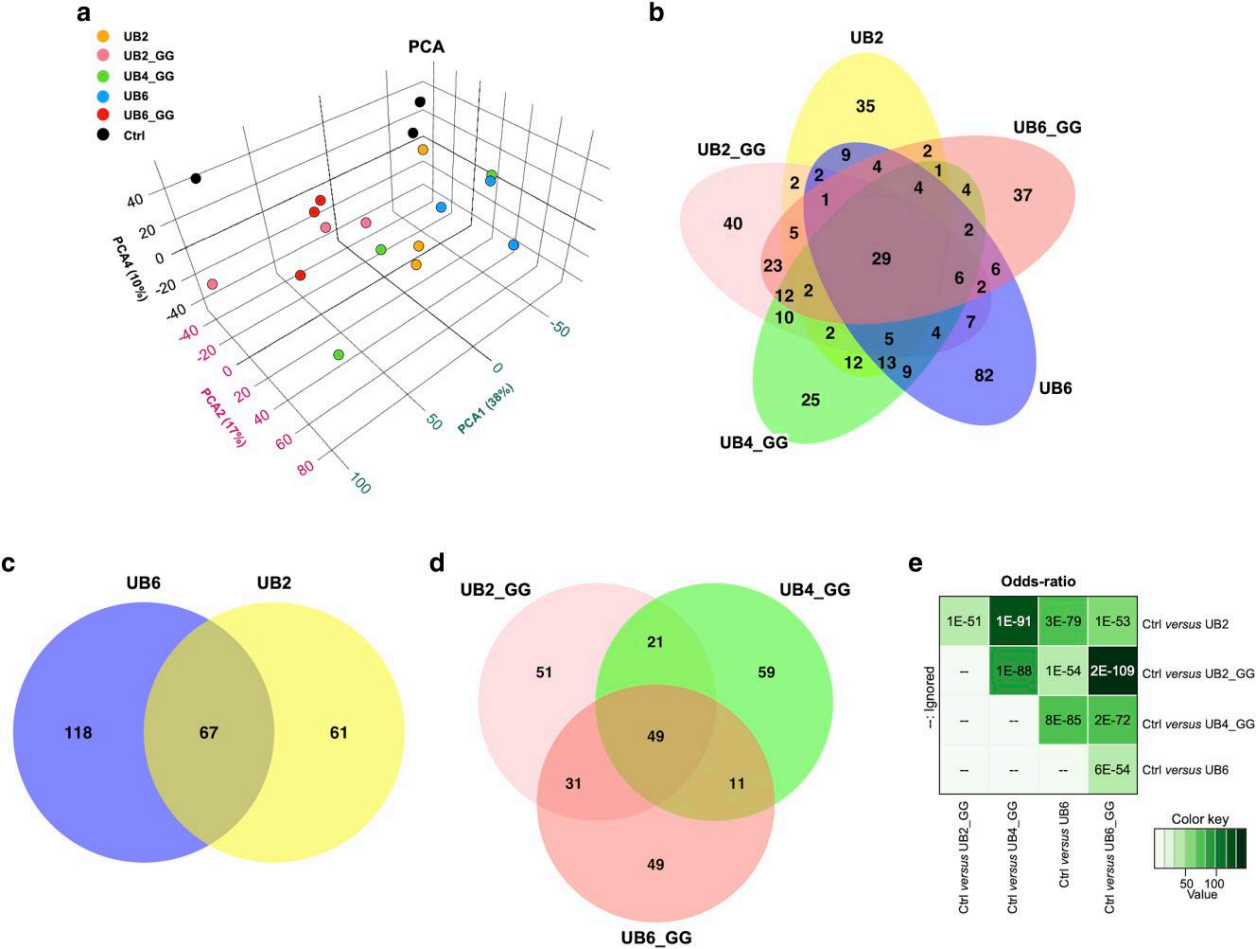
Principal component analysis (a), venn diagrams (b-d), and protein overlap with odds-ratio analyses (e) of the differentially abundant proteins relative to the control group observed between and among the different UBX groups, collectively indicating shared and distinct proteins among the groups.

Groups were tested in triplicate per genotype, with 20 flies per group. Approximately 100 µg protein per sample were used per group. The TMT tags used in each group are shown in Supplementary Fig. 2. We acquired 1,843,638 MS/MS spectra, identified 74,120 unique peptides corresponding to 7,907 unique proteins with a false discovery rate of <1%, and quantified 7,872 unique proteins with at least two spectral counts per protein. The Principal Component Analysis plot derived from these data is shown in Fig. 2a, providing a bird's-eye-view of similarities and differences among the assessed groups and replicates.

First, we compared all linear UB-expressing groups together to determine the extent to which the differentially abundant proteins in each group overlap with other constructs; we observed significant similarities among them (Fig. 2b-d). There are more extensive similarities between groups that have conjugation potential (UBX_GG) than the length of the UB chain (UB2 vs. UB2_GG, or UB6 vs. UB6_GG) (Fig. 2e).

Twenty-nine proteins were shared among all groups (Supplementary Fig. 3 lists their identities), compared to the control group (Fig. 2b). There was a unidirectional decrease in 13 proteins and an increase in 16 proteins across all groups (Fig. 2b and Supplementary Fig. 3). These included CYP4p1, CYP4ac, GstE5, and Ugt36E1, which are involved in detoxification and hormone metabolism (Gramates *et al.* 2022).

Each linear UB-expressing group also had between 25 and 82 proteins whose changes in abundance were unique to them (Fig. 2b). We further sub-categorized these groups into linear UB without or with a terminal “GG” motif. When comparing UB2 with UB6 lacking a terminal “GG”, there were approximately equal numbers (67 shared proteins; Fig. 2c and Supplementary Fig. 4), including a decrease in proteins involved in fatty acid metabolism, e.g. ACBP3 and ACBP5, which enable fatty Acyl-CoA free pool of long chain fatty acid accumulation in the cytosol (Gramates *et al.* 2022) as well as a decrease in enoyl-CoA hydratase (CG4594), which is involved in the breakdown of fatty acids via beta oxidation (Gramates *et al.* 2022). This was accompanied by a decrease in proteins predicted to enable ecdysteroid 22-kinase activity, as well as LSP2 which is inducible by ecdysteroids (Okamoto *et al.* 2009), and an increase in chitin and chitin interactors (CG13155 & CG32240). Furthermore, there was an upregulation of CG34717 (acyl-CoA reductase in *Drosophila*), which is predicted to function in long-chain fatty-acyl-CoA metabolic processes (Gramates *et al.* 2022). There were also differentially abundant proteins unique to each group (118 for UB6 and 61 for UB2; Fig. 2c).

When comparing the other linear UB chains that contain a terminal “GG” motif (Fig. 2d), we observed 49 shared proteins among all groups, including an increased abundance of CG11598 (lipase activity), cryptochrome (circadian feedback loop regulator) as well as a 2.5 log_2_FC increase in Ugt36E1 (involved in sex pheromone discrimination when expressed in the olfactory sensory neurons (Gramates *et al.* 2022)). Conversely, we identified a decrease in the protein levels of alpha-Est9 and CG4594 enoyl CoA hydratase (Fig. 2d and Supplementary Fig. 5).

The Venn diagrams in Fig. 2 indicate that there are distinct and shared proteomic changes when each of the transgenes encoding for linear UB chains is expressed ubiquitously in *Drosophila*. Odds-ratio analysis provides a statistical assessment of these overlaps (Fig. 2e), which indicates highly significant cross-regulation among the linear UB groups when compared to the control line. The degree of significance varies, with the highest overlap being observed between UB2 and UB4_GG, and the least between UB6 and UB6_GG. Collectively, these results demonstrate specific changes in the fly proteome caused by the expression of distinct linear UB chains. Supplementary Fig. 6 lists all the protein changes, compared to the control flies.

### Molecular and pathway analyses of the TMT proteomics data

**Fig. 3.**
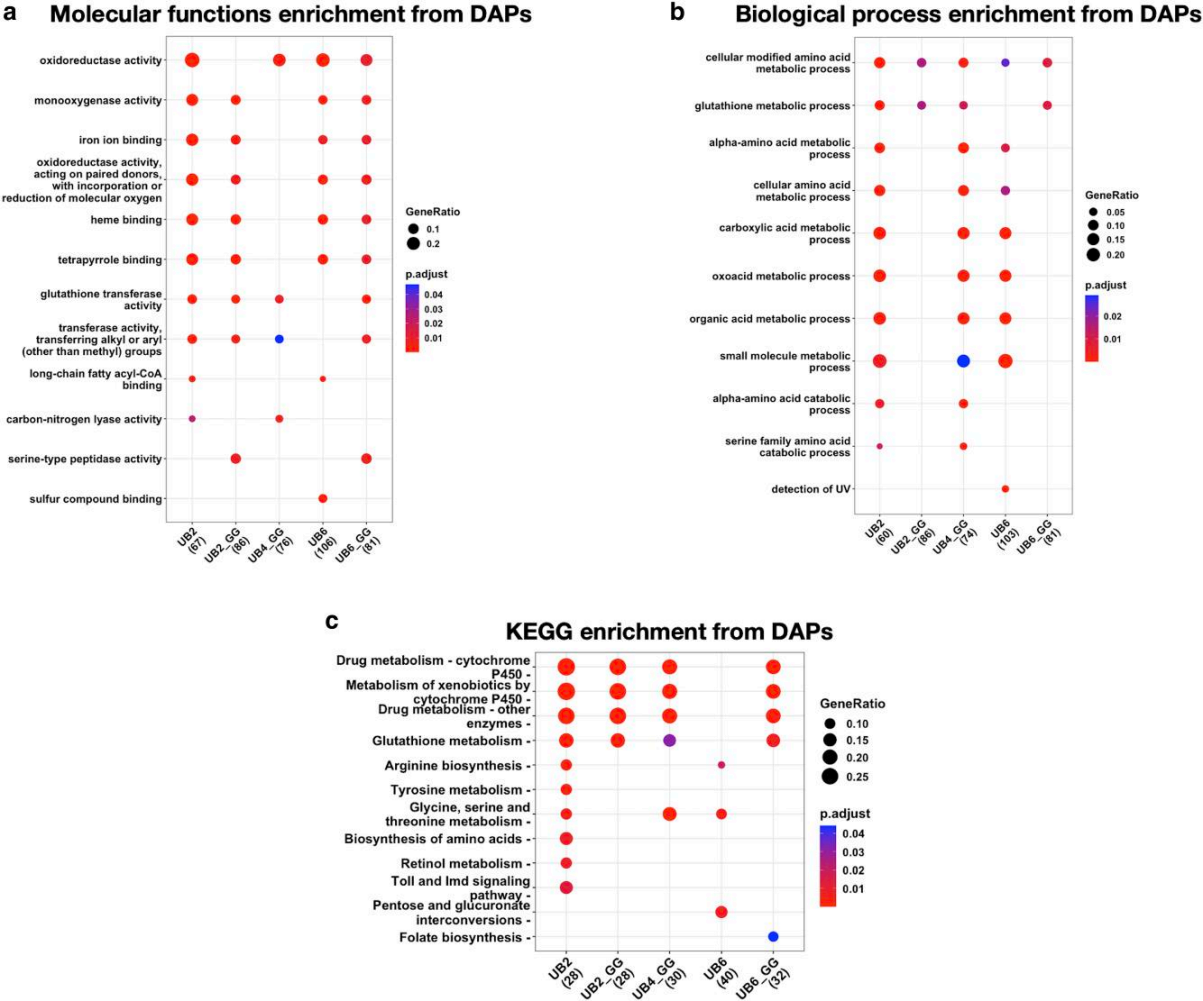
Gene ontology pathway enrichment analyses of differentially abundant proteins (DAPs) showing over-represented molecular functions (a), biological processes (b), and KEGG pathways (c) changed by the expression of specific linear UB species in flies. Key differences arise among the different groups expressing specific types of linear poly-UB, especially when comparing chains with a “GG” vs. not.

Pathway enrichment analyses of differentially abundant proteins highlighted key molecular functions and biological processes that were significantly altered by the expression of each of the linear UB species (Fig. 3). As shown in Fig. 3a, expression of each of the linear UB species led to up- or down-regulation of specific molecular functions in the fly. Some such changes (oxidoreductase activity, monooxygenase activity, iron ion binding, heme binding, tetrapyrrole binding, glutathione transferase activity, and transference of aryl and alkyl groups) were shared among 4 out of 5 of the different linear UB types. Expression of linear UB chains led to 4 or more (UB2_GG, UB4_GG, UB6) changes in functions, with UB2 and UB6_GG expression resulting in the largest number of such changes (10 and 9, respectively). Thus, expression of the linear UB chains of different lengths in the fly led to shared and distinct changes in molecular functions. When comparing UB chains without a terminal “GG” vs. poly-UB with a terminal “GG”, there was considerable overlap in the molecular functions modulated by UB chains, except for three pathways (oxidoreductase, long chain fatty acyl-CoA binding, and carbon-nitrogen activity) which are present in UB2 but absent in UB2_GG. UB2_GG, in turn, was enriched for serine-type peptidase activity while UB2 was not. Overall, UB4_GG displayed the fewest changes in molecular functions (Fig. 3a).

Overlaps and differences among the linear UB chains can be further contextualized when examining biological processes (Fig. 3b). UB2 and UB4_GG led to the largest number of changes in biological processes, a total of 10, mostly centered around energy metabolism, amino acid metabolism, and detoxification. UB2_GG and UB6_GG had the fewest, a total of 2 each, that were shared with UB2, UB4_GG, and UB6_GG (cellular modified amino acid metabolic process, glutathione metabolic process).

KEGG pathway enrichment also showcased UB2 (Fig. 3c) as the group with the most changed pathways (10), with the other groups varying from 3 (UB6) to 5 (UB4_GG and UB6_GG). Four pathways were shared among all groups, except for UB6: drug metabolism-cytochrome P450, metabolism of xenobiotics by cytochrome p450, drug metabolism-other enzymes, and glutathione metabolism. Four pathways were exclusive to UB2 (tyrosine metabolism, biosynthesis of amino acids, retinol metabolism, and Toll and IMD signaling pathway). UB6 had one pathway altered only in this group, pentose and glucuronate interconversions, whereas folate biosynthesis was altered only in UB6_GG. Collectively, these results again indicate shared and distinct processes influenced by linear UB of distinct lengths in *Drosophila*.

### Curated assessment of the proteomics data—the ubiquitin/proteasome system

**Fig. 4.**
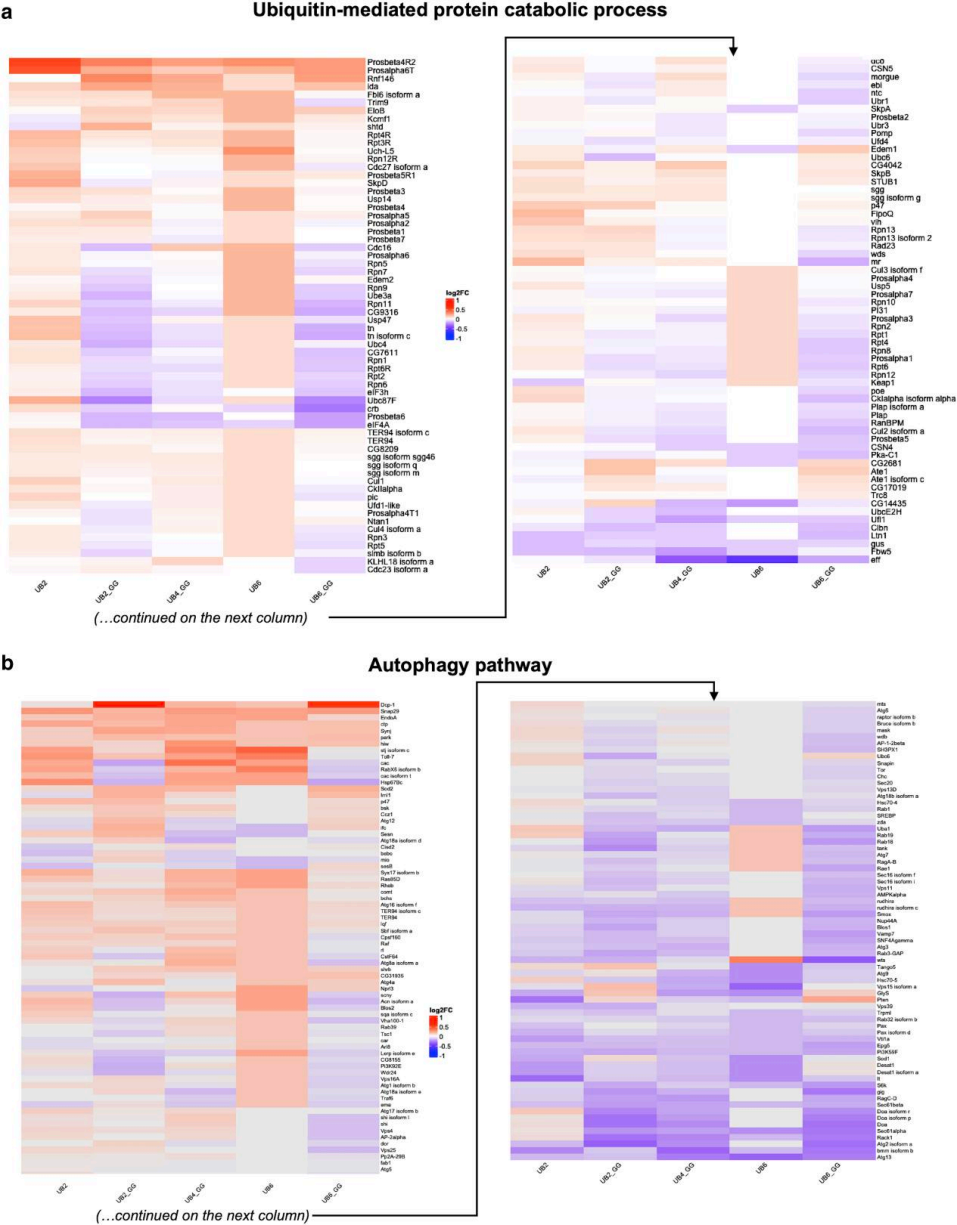
a) Heatmap of hierarchically clustered, TMT-quantified protein expression changes in curated UB-mediated catabolic processes in the presence of specific linear UB species with log2FC expression changes relative to the control group. b) Heatmap of proteins changes in the autophagy pathway in the presence of specific linear UB species. The levels of various proteasome-, UB-related enzymes and proteins, and autophagy-related components are impacted across the different genotypes.

We next took a curated look at processes generally linked to UB. We began by comparing “ubiquitin-mediated catabolic processes” among the five linear UB groups (Fig. 4a). The levels of many proteins were changed, albeit not markedly so overall. We observed that several 20S proteasome core alpha and beta subunits were increased across all linear UB groups, but to different extents depending on the exact component (the 20S core component is the degradative machinery of the proteasome (Goldberg *et al.* 2021)). We also observed changes in 19S proteasome cap subunits, including Rpn and Rpt proteins (the 19S regulatory cap component of the proteasome binds to, deubiquitinates, and unfolds substrates targeted for degradation (Goldberg *et al.* 2021)). In some cases, their levels were generally higher (e.g. Rpt3, Rpt4) and in other cases, they were lower in specific groups (e.g. Rpn9 in UB2_GG, UB6_GG) but higher in others (Ub2, UB6), potentially reflecting specific modifications to the functions of the 19S regulatory cap on a needs-basis, depending on the homeostatic status of the cell. Changes in the levels of various UB enzymes and UB-binding proteins were also detected across the different linear UB groups.

There were general trends that matched among UB2_GG, UB4_GG, and UB6_GG with swaths of proteins consistently regulated across these groups. These included various Rpn and Rpt proteins whose levels were lower, alpha and beta 20S subunits whose levels were higher, and some UB ligases and deubiquitinases whose levels also changed in the same direction within these groups. The combination of increased levels of some UB-dependent proteins vs. decreased levels of others could be an indication of the utilization of the UBX_GG linear chains in modulating cellular processes and pathways, presumably by targeting specific protein substrates in a manner dependent on the length of the available linear poly-UB.

There were also similarities between UB2 and UB6, whose patterns differed to some extent compared to the “GG” groups. In this case, most of the changes that reached significance were in the upregulated direction and included various proteasome subunits, UB ligases, deconjugases, and UB-binding proteins. We previously reported that UB6 is degraded in *Drosophila* by the proteasome in a manner dependent on the UB-binding proteins p47 and VCP (known in the fly as TER94) (Blount *et al.* 2018). The generally increased levels in proteasome subunits, UB ligases, and UB-binding proteins (Fig. 4a) are congruent with the notion of key roles being played by these proteins in the degradation of linear UB species, and of possible adaptations of the proteasome in response to changes in the abundance of linear UB chains. Altogether, these analyses indicate specific adaptations in the UB/proteasome system in *Drosophila* because of linear UB chains that can be conjugated onto other proteins vs. UB chains that cannot be conjugated.

### Curated assessment of hierarchical proteomics data—the autophagy pathway

We next examined the hierarchical clustering of protein changes involved in the autophagy pathway, where UB signaling is also important and includes the utilization of linear UB chains (Blount, Johnson, *et al.* 2020). As with the other pathways, there were similarities and differences among the different linear UB groups (Fig. 4b). The “GG” groups trended more similarly in their overall patterns, especially UB2_GG and UB6_GG. UB2 and UB6 shared some similarities, especially in the bottom one-third of Fig. 4b, but not as much in other portions. A few proteins stood out for the changes in their levels among the different groups. From the top, Dcp-1 (Death caspase-1, involved in nonapoptotic processes, including autophagy (Gramates *et al.* 2022)) was upregulated in the presence of UB2_GG and UB6_GG, with minimal or no upregulation in the other groups. Hsp67Bc (a small heat shock protein that stimulates autophagy (Gramates *et al.* 2022)) had decreased levels with UB2_GG and UB6_GG, but increased levels with the other groups. Another interesting example was wts (warts, a kinase involved in Hippo signaling and tissue growth (Gramates *et al.* 2022)), whose levels were increased with UB6, but decreased with UB6_GG. Lastly, the levels of Atg13 (Autophagy-related 13, which controls the initiation of autophagosome formation (Gramates *et al.* 2022)) were lower among all groups, but more so with the longer UB chains. Other examples with differences in their levels among groups exist, including Sod1, gig, Vps15, Pten, Atg2, etc. Once again, it appears that linear poly-UB with different lengths and conjugation capabilities influences a key pathway, in this case autophagy, in specific ways in the fruit fly. In the case of the “GG” groups, the decrease in protein levels is presumably a sign of the conjugation of these protein substrates to UB2_GG, UB4_GG, or UB6_GG, which would target them for degradation by the autophagy/lysosome or UB/proteasome systems. Conversely, the increased levels of other proteins may reflect their increased stabilization via ubiquitination.

### Curated assessment of hierarchical proteomics data—the immune pathway

**Fig. 5.**
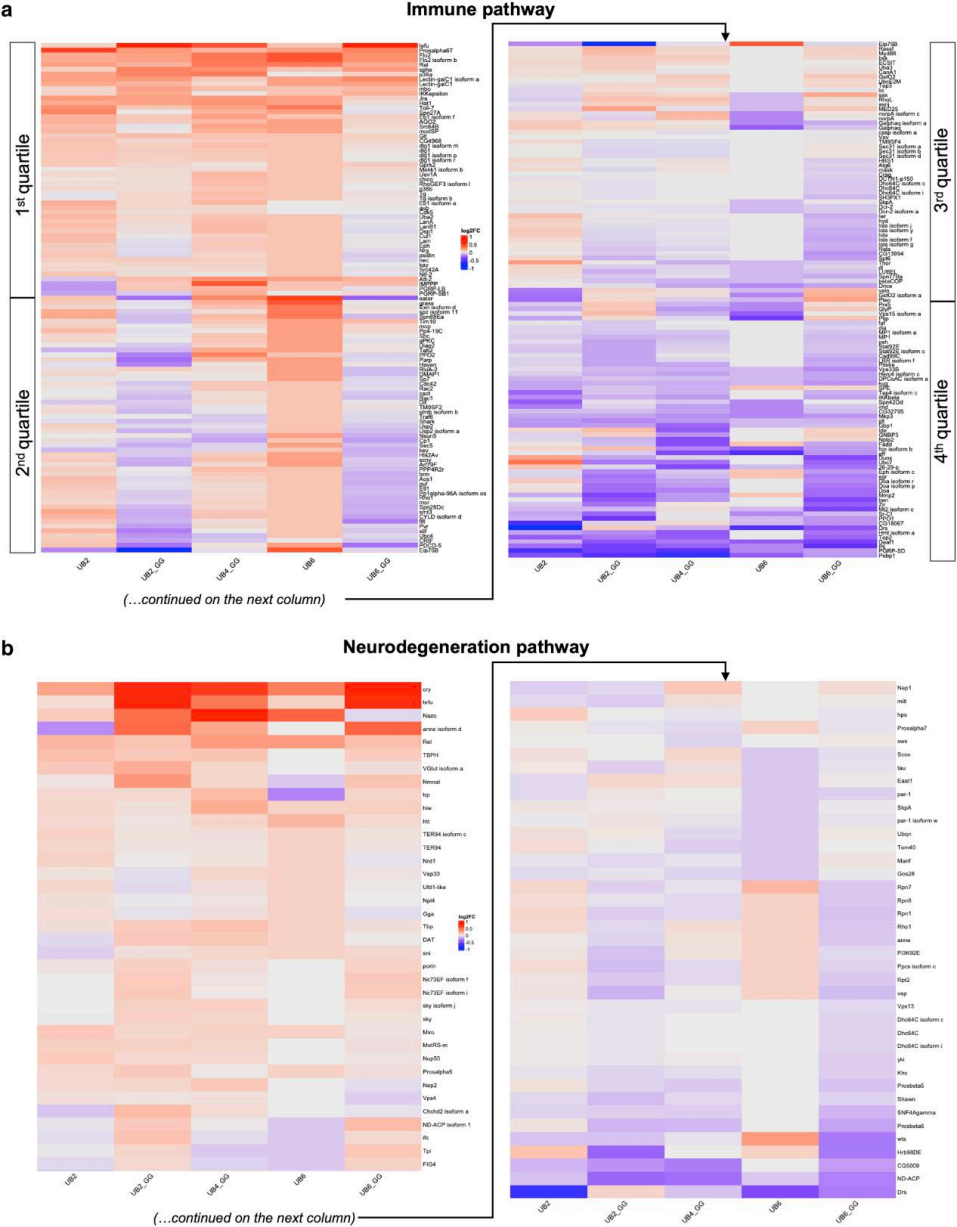
a) Heatmap of hierarchically clustered, TMT-quantified protein expression changes in curated immune pathway in the presence of specific linear UB chains. Similarities and differences among the groups fall roughly into 4 areas, highlighted as “quartiles”. b) Heatmap of hierarchically clustered, TMT-quantified protein expression changes in curated neurodegeneration pathway in the presence of specific linear UB species. Similarities and differences are evident when comparing linear UB without or with a terminal “GG”.

Since linear UB is closely involved in the immune response (Blount, Johnson, *et al.* 2020), we next focused on changes in immune pathways, represented in a hierarchical heatmap in Fig. 5a. While there was overlap in the protein levels and their change in direction among the different linear UB groups (e.g. top quartile for UB2, UB2_GG, UB4_GG, UB6, and UB6_GG), differences in patterns also emerged. For example, in the second quartile of Fig. 5, UB2_GG and UB6_GG were more similar to each other, and UB6 stood out by itself in the directionality of change, generally higher. In the third quartile, UB2 was less similar than the others, whereas in the bottom quartile UBX_GG were more similar among each other, and UBX were more similar between themselves.

Among the many proteins identified, some stood out in terms of their extent and directionality of change. From the top, one striking example was the protein tefu (telomere fusion, a nonspecific serine/threonine kinase (Gramates *et al.* 2022)), whose levels were upregulated in the presence of UB2_GG, UB6_GG, and less markedly so in UB4_GG, but were not altered as much in the non “GG” groups. Another interesting case was eater (a transmembrane receptor involved in the phagocytosis of Gram-positive bacteria (Gramates *et al.* 2022)), whose levels were reduced in the presence of UB2_GG and UB6_GG, but increased to varying extents in the other groups. Other similar examples (e.g. PDCD-5, Galphaq, eff, Drs, etc.) collectively suggest distinct outcomes on immune proteins resulting from linear UB chains with different lengths and ability to become conjugated onto other proteins in *Drosophila*.

### Curated assessment of hierarchical proteomics data—the neurodegeneration pathway

Lastly, since UB-mediated protein quality control is a bona fide protective molecular mechanism in neurodegenerative diseases (Todi and Paulson 2011; Azari *et al.* 2022; Thellung *et al.* 2022; Whittemore *et al.* 2022; Suresh *et al.* 2023), we assessed changes to neurodegeneration pathway proteins upon expression of the UBX and UBX_GG constructs (Fig. 5b). Although the linear UB constructs were ubiquitously expressed and not targeted to the nervous system, we identified changes in the abundance of proteins that are neuroprotective in distinct neurodegenerative pathologies, most notably, cry, tefu, and Nazo. Cry (cryptochrome; a regulator of the circadian rhythm (Gramates *et al.* 2022)) was upregulated across all species, but especially in the presence of UBX_GG. Tefu (telomere fusion, a nonspecific serine/threonine kinase whose human orthologue, when mutated, causes ataxia telangiectasia (Gramates *et al.* 2022)) was significantly increased in UB2_GG and UB6_GG. Similarly, Nazo (involved in triglyceride homeostasis (Gramates *et al.* 2022)) was differentially increased in UB2_GG, UB4_GG, and UB6 with its changes in UB2 and UB6_GG falling below the filtering cutoff (please see Supplementary Fig. 6 and the raw data spreadsheet). Overall, UB2_GG and UB6_GG shared patterns of changes across the board. UB6 stands apart from the other groups, and there are some trends of similarity between UB2 and UB4_GG. Future studies focusing on the nervous system with these specific UB species may reveal insights into the role of linear UB in brain function, and how chains of specific length may influence the initiation and progression of neurodegeneration.

### Decreased protein levels in flies expressing linear UB with a terminal “GG” motif

As shown in Figs. 1d, f and g, and as we have published before with UB6_GG (Blount *et al.* 2018), expression of linear UB species with a terminal “GG” motif leads to higher molecular weight ubiquitin smears. We do not observe this type or extent of migration with UB2, which lacks a terminal “GG” (Fig. 1d); we have also shown before that smears of UB6, which also lacks a terminal “GG”, result from conjugation of endogenous UB onto these noncleavable, nonconjugatable chains (summarized in Fig. 1b; (Blount *et al.* 2018; Blount, Libohova, *et al.* 2020)). UB6_GG can be conjugated onto endogenous proteins in the fly and in mammalian cell culture (Blount *et al.* 2018). Thus, we reasoned that the higher molecular species that we observe with the “GG”-containing linear UB likely include endogenous proteins onto which UB2_GG, UB4_GG, or UB6_GG was conjugated, a modification that could impact their levels.

**Fig. 6.**
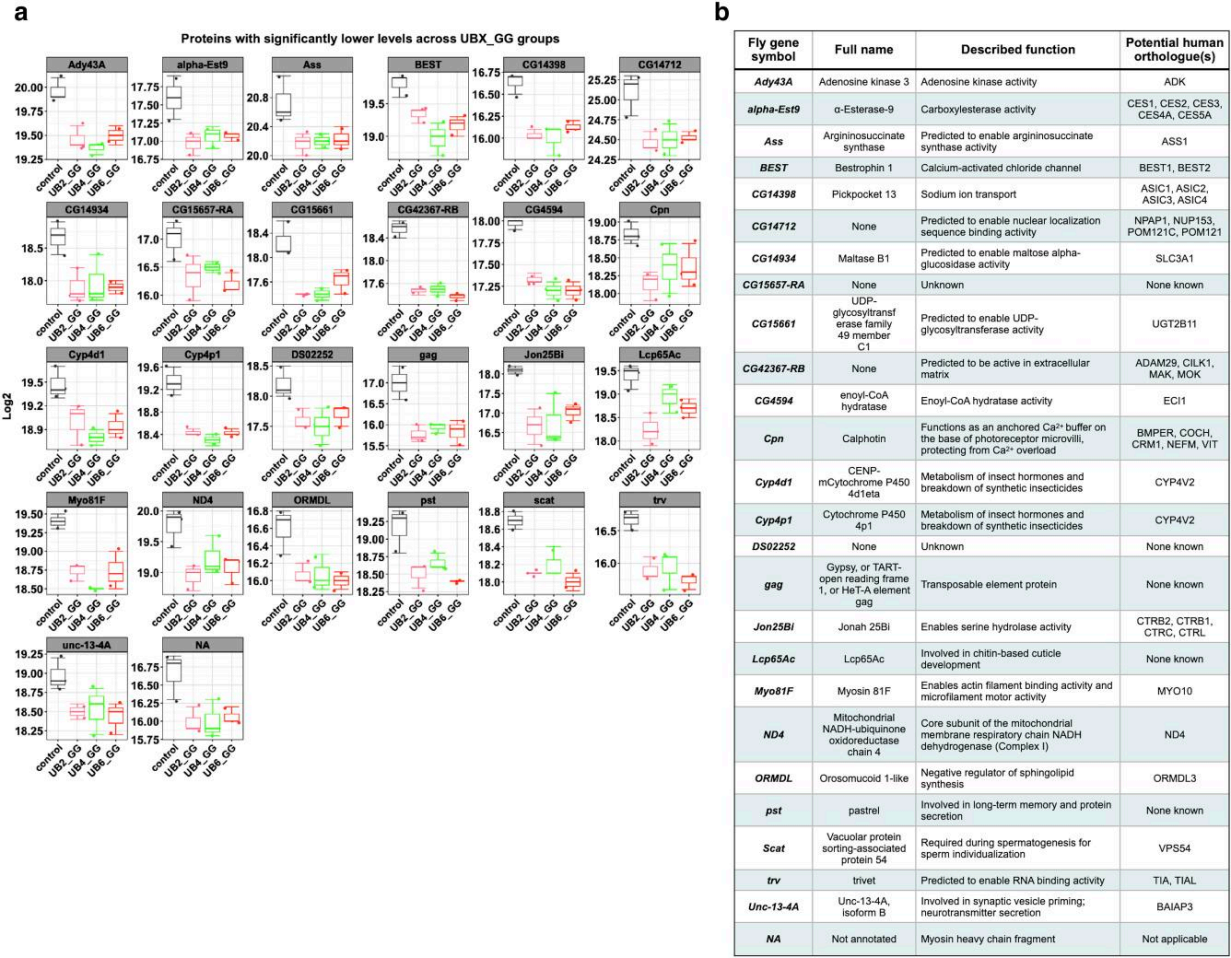
Boxplot of log2 differentially abundant protein expression changes (a; mean ± SEM) and their known orthologues and functions (b) whose levels were consistently lower in the presence of UBX_GG. Gene name symbol, reported function, and potential human orthologues were sourced from flybase.org (Gramates *et al.* 2022). When suitable, based on “Best Score” outputs, multiple potential orthologues are shown. For panel (a), the individual data points shown represent biological replicates from *N* = 3.

Since UB is commonly thought of as a degradative signal, we curated a list of proteins whose levels were consistently decreased among the three groups of linear UB chains with a “GG” motif. As summarized in Fig. 6, the levels of 26 proteins were significantly reduced in the presence of UB2_GG, UB4_GG, and UB6_GG. Some of them have known functions that span different activities (transcription, cytoskeletal, etc.; potential human orthologues in Fig. 6b were sourced from (Gramates *et al.* 2022)), whereas a few do not have a known function. According to the STRING database, Metascape, and FlyEnrichr, no clear pathways or processes emerge from these hits (e.g. Supplementary Fig. 7). Altogether, these findings indicate that linear UB species of different length may share common protein targets.

## Discussion

Ubiquitination is a fundamental posttranslational modification that controls the function and levels of target proteins. Recently, linear UB chains released from ubiquitinated proteins have emerged as an important form of UB assembly that may play regulatory roles for many cellular processes, such as immune responses and proteostasis (Kirisako *et al.* 2006; Inn *et al.* 2011; Rivkin *et al.* 2013; Shimizu *et al.* 2015; Blount, Johnson, *et al.* 2020; Tokunaga and Ikeda 2022; Gao *et al.* 2023; Sasaki and Iwai 2023; Sato *et al.* 2023). Our previous studies in *Drosophila melanogaster* demonstrated the versatility of transgene-expressed linear poly-UB chains that cannot be dismantled into single UB moieties, which can be conjugated to target proteins or, alternatively, can be themselves ubiquitinated and degraded (Blount *et al.* 2019; Johnson *et al.* 2019; Blount, Johnson, *et al.* 2020; Blount, Libohova, *et al.* 2020).

Here, we set to determine what protein substrates are modulated by linear UB chains. For this purpose, we examined the changes in protein levels induced by noncleavable, linear UB chains comprising 2, 4, or 6 UB when expressed ubiquitously in *Drosophila*. We found numerous proteins—and corresponding processes and pathways—whose levels were changed in the same or opposite direction by the linear UB chains with distinct lengths and capacity for ligation onto target proteins. Collectively, we found similarities in the proteins and associated cellular functions in the presence of linear UB chains lacking a terminal “GG” vs. the linear UB chains with “GG”. Differences also emerged in the proteins and cellular processes impacted by linear chains with terminal “GG” motifs but consisting of different UB lengths. We interpret these results to indicate that linear UB species of distinct lengths have specific roles in vivo in *Drosophila*, and that they have the potential to modulate a wide range of cellular activities.

To obtain enough material for TMT mass spectrometry, we utilized a ubiquitous Gal4 to drive the transgenic expression of linear poly-UB in *Drosophila* throughout development and adulthood. While this analysis provides insight into the proteome subsets that are generally and consistently regulated by linear UB chains across multiple tissues, it is possible that the modality of their regulation differs in specific tissues. Consequently, it is likely that many more proteins are regulated by linear UB chains in specific tissues and that such tissue-specific targets might be missed by the current analysis of organism-wide changes. Likewise, the proteins modulated by linear UB chains may also change depending on environmental stimuli (e.g. diets and infections) and settings (e.g. aging vs. development). Although this study utilized male flies, it is possible that linear UB chains differentially modulate the proteome in males vs. females and that they regulate sex-specific processes such as reproduction in female flies. Nonetheless, despite the limitations of the current analysis, this study provides fundamental insight into the proteins that are generally modulated by linear UB chains and thus enhances our understanding of how this component of the ubiquitin-proteasome systems contributes to maintaining cellular homeostasis.

Our findings are likely to be widely applicable to eukaryotes. The UB system is strongly conserved from yeast to humans (Komander and Rape 2012). This conservation is even more pronounced between *Drosophila* and humans at the level of E1, E2, E3, and DUBs, and also in relation to proteins and pathways controlled by UB (Komander *et al.* 2009; Williamson *et al.* 2009; Clague *et al.* 2012; Komander and Rape 2012; Tsou *et al.* 2012; Clague *et al.* 2013; Liang *et al.* 2015; Clague *et al.* 2019; Aalto *et al.* 2024). Linear UB chains are evolutionarily preserved, and therefore our findings in *Drosophila* contribute to the general understanding of the fundamental role of linear UB chains in reshaping the proteome. One such example underscored by our current data is the regulation of circadian rhythms by the UB system. Circadian rhythms are driven by a transcriptional feedback loop based on CLOCK and BMAL1 (known as “cycle” in the fly) proteins, which induce the transcription of their inhibitors, Period (PER) and Cryptochrome (CRY). The activity of CLOCK-BMAL1 is then re-established via the UB-mediated degradation of PER and CRY (Takahashi 2017), which is mediated by the E3 ubiquitin ligase FBXL3 (Shi *et al.* 2013). Therefore, our findings that linear UB chains modulate fly cryptochrome protein levels indicate that linear UB contributes to the UB-dependent regulation of circadian clocks (Srikanta and Cermakian 2021).

We started this study to further enhance our understanding of the flexibility of the UB system in vivo. We had originally hypothesized that the expression of linear UB that cannot be dismantled into its constituent moieties is toxic to organisms (Blount *et al.* 2018). We found that this was not the case, unless the ability of the cell to manipulate the linear chain was severely restricted—meaning that it would not be able to cleave it, conjugate it to another protein, or conjugate UB onto it (Blount *et al.* 2018; Blount, Libohova, *et al.* 2020). We also found that exogenous, linear UB that could not be disassembled but was able to be conjugated onto other proteins, could indeed do so in vivo (Blount *et al.* 2018). Because these data challenged the original hypothesis that linear, free UB chains would be toxic, we next wondered how the cell might employ linear UB chains of different lengths, and how these chains may influence cellular processes and homeostasis. As indicated by the quantitative proteomics analyses that we report in this study, it appears that *Drosophila* cells can utilize distinct linear UB chains to modulate many important cellular processes, thus providing insight into the possible roles that endogenous linear UB chains may play in vivo.

In conclusion, we presented evidence that linear UB chains remodel the proteome and influence the levels of hundreds of important regulators of homeostasis in *Drosophila*.

## Supplementary Material

jkae209_Supplementary_Data

## Data Availability

All data related to this manuscript are present in the figures and Supplementary Files associated with this manuscript. The mass spectrometry proteomics data have been deposited to the ProteomeXchange Consortium via the PRIDE partner repository with the dataset identifier PXD052020. Supplementary material available at G3 online.
