# Supplementary material for: Linear poly-ubiquitin remodels the proteome and influences hundreds of regulators in Drosophila
Source: G3 (Bethesda). 2024 Sep 26;14(11):jkae209. doi: 10.1093/g3journal/jkae209 (PMC11540324; doi:10.1093/g3journal/jkae209)

Supplemental figure 1

Un-cropped blots. For each panel, images are organized left-to-right as in the main figures

Figure 1D

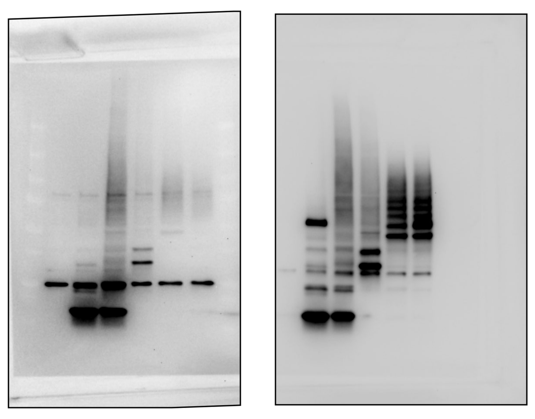

Figure 1E

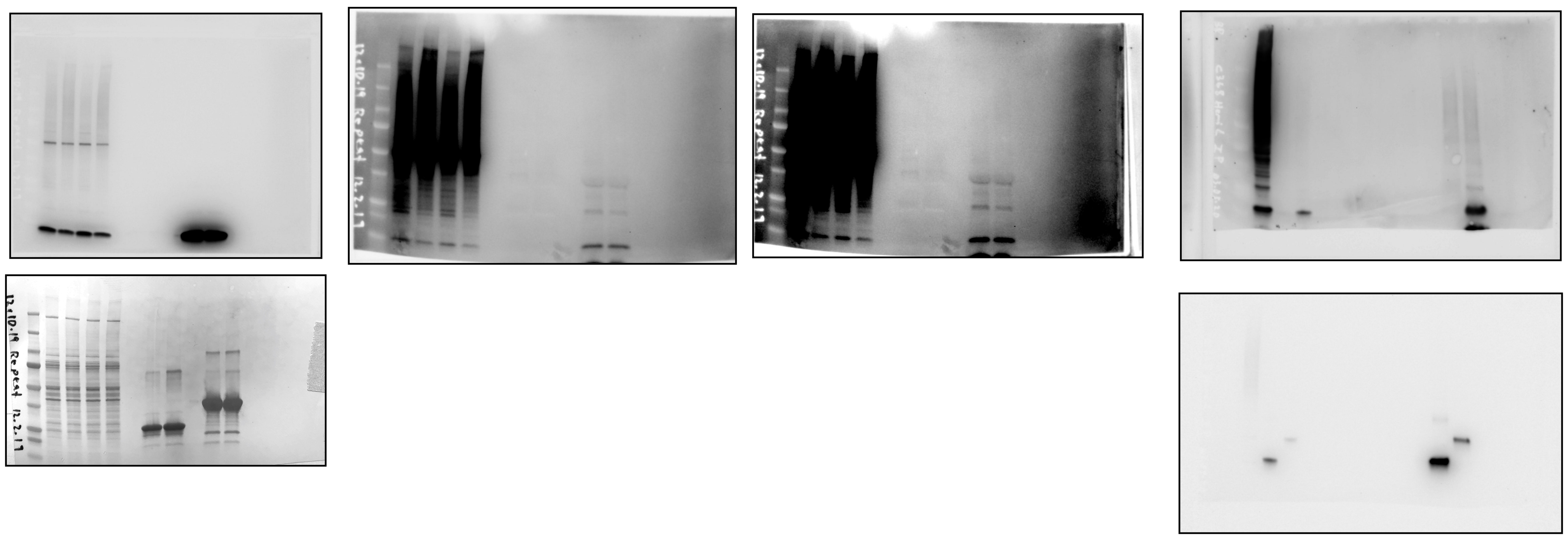

Figure 1F

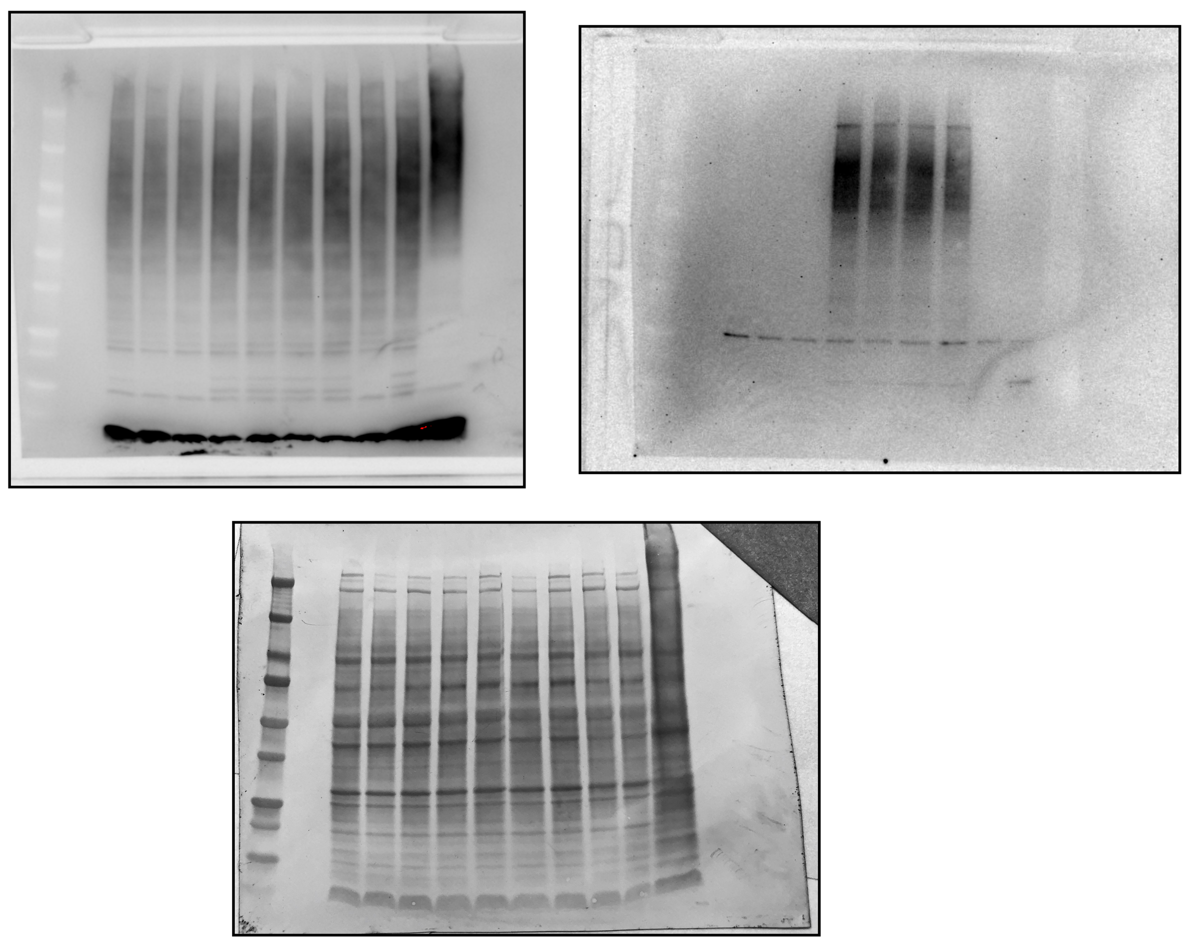

Figure 1G

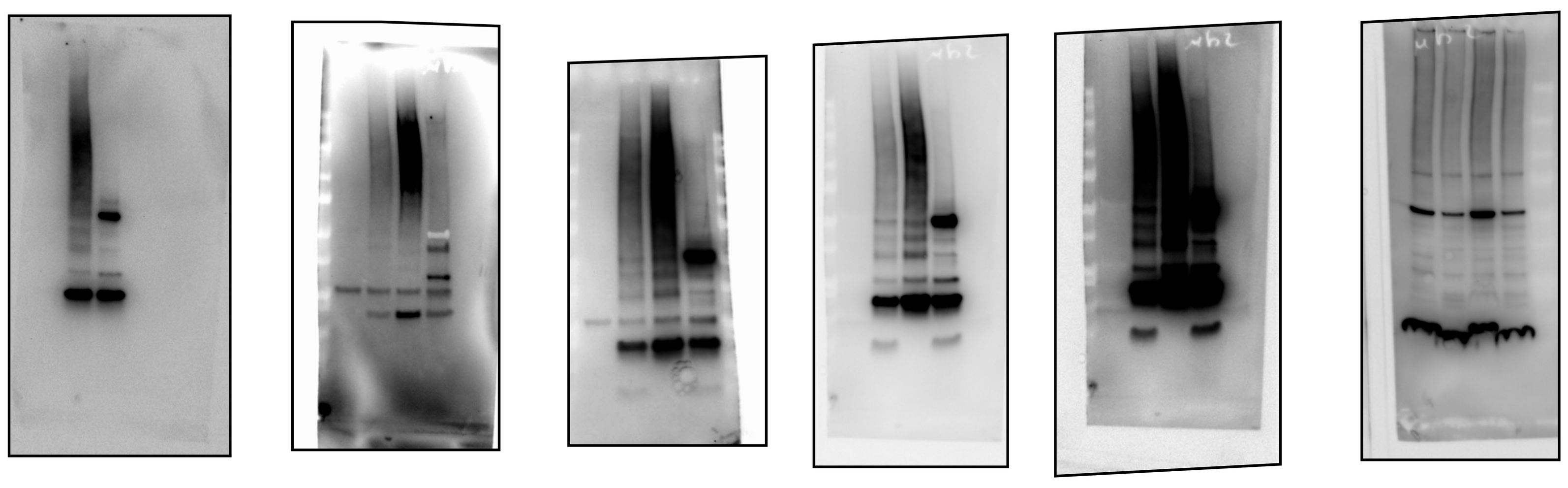

Supplemental figure 2

Samples (Group)	TMT Tags
S1. **Ctrl** flies (sqh-Gal4)	126
S2. **Ctrl** flies (sqh-Gal4)	127N
S3. **Ctrl** flies (sqh-Gal4)	127C
S4. sqh-Gal4>**Ub2**	128N
S5. sqh-Gal4>**Ub2**	128C
S6. sqh-Gal4>**Ub2**	129N
S7. sqh-Gal4>**Ub2GG**	129C
S8. sqh-Gal4>**Ub2GG**	130N
S9. sqh-Gal4>**Ub2GG**	130C
S10. sqh-Gal4>**Ub4GG**	131N
S11. sqh-Gal4>**Ub4GG**	131C
S12. sqh-Gal4>**Ub4GG**	132N
S13. sqh-Gal4>**Ub6**	132C
S14. sqh-Gal4>**Ub6**	133N
S15. sqh-Gal4>**Ub6**	133C
S16. sqh-Gal4>**Ub6GG**	134N
S17. sqh-Gal4>**Ub6GG**	134C
S18. sqh-Gal4>**Ub6GG**	135N

Supplemental figure 2: TMT tags used for each group.

Supplemental figure 3

	Protein accession	Protein name	UB2	UB2_GG	UB4_GG	UB6	UB6_GG
1	sp|O97069|ASSY_DROME	Ass	-0.4825814	-0.5535982	-0.5781328	-0.8	-0.5193034
2	sp|P04146|COPIA_DROME	GIP	0.68746516	1.03308202	0.57314505	1.3	0.60869887
3	sp|P20828|GAG2_DROME	gag	-1.2970645	-1.2239952	-1.0376151	-1.3	-1.1001391
4	sp|P33269|CP4D1_DROME	Cyp4d1	-0.6119423	-0.4571317	-0.6557903	-0.8	-0.5076349
5	sp|Q8I7Q0|GAGO_DROME	gag	1.00688961	0.69128298	0.78282383	1.2	0.73424036
6	sp|Q9V558|CP4P1_DROME	Cyp4p1	-1.1085153	-0.8989049	-1.0312184	-0.9	-0.9092534
7	sp|Q9V773|C6A20_DROME	Cyp6a20	0.59772225	0.58263226	0.57946412	0.8	0.47663296
8	sp|Q9VMS9|C4AC1_DROME	Cyp4ac1	0.71586911	0.79265398	0.6834434	0.9	0.90848007
9	tr|A0A0A1EI90|A0A0A1EI90_DROME	Myo81F	-0.7106082	-0.6467478	-0.8899753	-1.1	-0.668474
10	tr|C9QP70|C9QP70_DROME	CG43402	0.49153017	0.49334586	0.53745674	0.7	0.60408381
11	tr|D5SHN8|D5SHN8_DROME	CG42367-RB	-0.8856149	-1.113475	-1.0704593	-1	-1.1946644
12	tr|E1JIZ9|E1JIZ9_DROME	trv	-0.6523732	-0.7536639	-0.740729	-0.8	-0.9494164
13	tr|Q3HKQ6|Q3HKQ6_DROME	CG11598	1.03439087	1.89191623	1.63812239	1.3	1.85407939
14	tr|Q4V512|Q4V512_DROME	CG14934	-0.759142	-0.7965781	-0.7398368	-0.5	-0.7927147
15	tr|Q4V5F7|Q4V5F7_DROME	CG13155	2.72676229	2.33758938	2.46680952	2.6	2.538089
16	tr|Q59DY1|Q59DY1_DROME	CG14398	-0.6523821	-0.576575	-0.6301862	-0.6	-0.4835909
17	tr|Q5BI69|Q5BI69_DROME	GstE5	0.56328352	0.79626741	0.68652293	0.7	0.78905782
18	tr|Q7JVX3|Q7JVX3_DROME	BEST	-0.5720014	-0.4676864	-0.8157421	-0.5	-0.6082346
19	tr|Q7JWW6|Q7JWW6_DROME	Ady43A	-0.7545589	-0.5372475	-0.601009	-0.6	-0.4563347
20	tr|Q7KK90|Q7KK90_DROME	GstE1	0.48503298	0.55916885	0.53690798	0.6	0.74263634
21	tr|Q8SZG4|Q8SZG4_DROME	Jon25Bi	-0.9577865	-1.4739568	-1.4120246	-0.8	-1.0673406
22	tr|Q8T0X2|Q8T0X2_DROME	GstE8	0.49168534	0.76609698	0.45863236	0.5	0.68508131
23	tr|Q8T3S2|Q8T3S2_DROME	CG5539	0.8816106	0.77832252	0.78498925	0.8	0.90982403
24	tr|Q8T8R2|Q8T8R2_DROME	GstE8	0.49168534	0.76609698	0.45863236	0.5	0.68508131
25	tr|Q8T8W0|Q8T8W0_DROME	CG2127	0.57103239	0.71503155	0.64138251	0.5	0.71140192
26	tr|Q9VJ45|Q9VJ45_DROME	Ugt36E1	2.30669086	2.25270821	2.29945175	2.4	2.30766297
27	tr|Q9VL67|Q9VL67_DROME	CG4594	-0.7724872	-0.6417236	-0.7811471	-0.6	-0.7577288
28	tr|Q9VLU3|Q9VLU3_DROME	CG12560	1.84897078	1.70729399	1.73194772	1.9	1.59826612
29	tr|Q9VVZ4|Q9VVZ4_DROME	CG14095	0.52355152	0.46595864	0.66956141	0.8	0.69399909

Supplemental figure 3: List of proteins intersecting in figure 2A and their directionality of change.

Supplemental figure 4

	Protein accession	Protein name	UB2	UB6
1	sp|O97069|ASSY_DROME	Ass	-0.4825814	-0.8
2	sp|P04146|COPIA_DROME	GIP	0.68746516	1.3
3	sp|P20828|GAG2_DROME	gag	-1.2970645	-1.3
4	sp|P23779|CYTL_DROME	Cys	-0.5952552	-0.8
5	sp|P33269|CP4D1_DROME	Cyp4d1	-0.6119423	-0.8
6	sp|Q24388|LSP2_DROME	Lsp2	-1.6808086	-0.7
7	sp|Q8I7Q0|GAGO_DROME	gag	1.00688961	1.2
8	sp|Q9V558|CP4P1_DROME	Cyp4p1	-1.1085153	-0.9
9	sp|Q9V773|C6A20_DROME	Cyp6a20	0.59772225	0.8
10	sp|Q9VLF6|U585_DROME	CG18661	0.64862372	0.7
11	sp|Q9VMS9|C4AC1_DROME	Cyp4ac1	0.71586911	0.9
12	tr|A0A0A1EI90|A0A0A1EI90_DROME	Myo81F	-0.7106082	-1.1
13	tr|B9EQU3|B9EQU3_DROME	Sfp24Ba	-0.9449799	-1.1
14	tr|B9ER21|B9ER21_DROME	CG12224-RA	-0.5056706	-0.5
15	tr|C4NAP3|C4NAP3_DROME	Sfp38D	-0.797258	-0.7
16	tr|C9QP70|C9QP70_DROME	CG43402	0.49153017	0.7
17	tr|D5SHN8|D5SHN8_DROME	CG42367-RB	-0.8856149	-1
18	tr|D5SHT6|D5SHT6_DROME	CG5707-RA	-0.5848641	-0.5
19	tr|E1JIZ9|E1JIZ9_DROME	trv	-0.6523732	-0.8
20	tr|F6J907|F6J907_DROME	CG3926	-0.5136025	-0.7
21	tr|H0RNA2|H0RNA2_DROME	CG18180-RA	-0.4530459	-0.5
22	tr|Q059A3|Q059A3_DROME	CG8515	0.54394453	0.5
23	tr|Q3HKQ6|Q3HKQ6_DROME	CG11598	1.03439087	1.3
24	tr|Q4V512|Q4V512_DROME	CG14934	-0.759142	-0.5
25	tr|Q4V5F7|Q4V5F7_DROME	CG13155	2.72676229	2.6
26	tr|Q59DY1|Q59DY1_DROME	CG14398	-0.6523821	-0.6
27	tr|Q5BI69|Q5BI69_DROME	GstE5	0.56328352	0.7
28	tr|Q5BII9|Q5BII9_DROME	CG6415	-0.5364731	-0.6
29	tr|Q5U189|Q5U189_DROME	Argl	-0.5211512	-0.7
30	tr|Q6IJM7|Q6IJM7_DROME	HDC14603	-0.7255384	-0.6
31	tr|Q7JVX3|Q7JVX3_DROME	BEST	-0.5720014	-0.5
32	tr|Q7JWW6|Q7JWW6_DROME	Ady43A	-0.7545589	-0.6
33	tr|Q7K025|Q7K025_DROME	CG11892	-0.7896885	-0.8
34	tr|Q7KK90|Q7KK90_DROME	GstE1	0.48503298	0.6
35	tr|Q8I0L0|Q8I0L0_DROME	CG32240	0.61308295	0.6
36	tr|Q8IRD6|Q8IRD6_DROME	Drsl4	-2.1484347	-1.7
37	tr|Q8MZ52|Q8MZ52_DROME	CG10514	-0.6624381	-0.7
38	tr|Q8SY29|Q8SY29_DROME	CG18180	-0.4519178	-0.5
39	tr|Q8SZG4|Q8SZG4_DROME	Jon25Bi	-0.9577865	-0.8
40	tr|Q8T0X2|Q8T0X2_DROME	GstE8	0.49168534	0.5
41	tr|Q8T3S2|Q8T3S2_DROME	CG5539	0.8816106	0.8
42	tr|Q8T444|Q8T444_DROME	Argl	-0.839496	-0.9
43	tr|Q8T8R2|Q8T8R2_DROME	GstE8	0.49168534	0.5
44	tr|Q8T8W0|Q8T8W0_DROME	CG2127	0.57103239	0.5
45	tr|Q960C6|Q960C6_DROME	Argl	-0.8656419	-1
46	tr|Q961N0|Q961N0_DROME	alpha-Est2	-0.8046219	-0.8
47	tr|Q9VAU2|Q9VAU2_DROME	Sid	0.67463337	0.7
48	tr|Q9VBT6|Q9VBT6_DROME	CG10514	-0.6894322	-0.8
49	tr|Q9VBV8|Q9VBV8_DROME	Alg9	-1.0933552	-1.2
50	tr|Q9VE10|Q9VE10_DROME	CG14285	0.48713683	0.5
51	tr|Q9VES5|Q9VES5_DROME	CT34717	1.15886255	1.1
52	tr|Q9VGF3|Q9VGF3_DROME	CG18547	-0.5012899	-0.5
53	tr|Q9VGK3|Q9VGK3_DROME	CG14715	-0.6661181	-0.7
54	tr|Q9VGT0|Q9VGT0_DROME	Ugt35B1	-0.9537272	-0.9
55	tr|Q9VJ45|Q9VJ45_DROME	Ugt36E1	2.30669086	2.4
56	tr|Q9VJI8|Q9VJI8_DROME	ChLD3	-1.7489446	-1.4
57	tr|Q9VL67|Q9VL67_DROME	CG4594	-0.7724872	-0.6
58	tr|Q9VLU3|Q9VLU3_DROME	CG12560	1.84897078	1.9
59	tr|Q9VMM2|Q9VMM2_DROME	CG11034	-0.8240347	-0.9
60	tr|Q9VS22|Q9VS22_DROME	Acbp3	-0.8729231	-1.3
61	tr|Q9VSP9|Q9VSP9_DROME	Acbp5	-0.6227762	-0.7
62	tr|Q9VUW1|Q9VUW1_DROME	cg17026	0.92522968	1.2
63	tr|Q9VV23|Q9VV23_DROME	CG13044	0.99336732	0.6
64	tr|Q9VV30|Q9VV30_DROME	BcDNA	0.63182135	0.7
65	tr|Q9VVZ4|Q9VVZ4_DROME	CG14095	0.52355152	0.8
66	tr|Q9W043|Q9W043_DROME	HL03644	-0.5848641	-0.5
67	tr|Q9W3L4|Q9W3L4_DROME	CG2233	-0.8454788	-0.8

Supplemental figure 4: List of proteins intersecting in figure 2B and their directionality of change.

Supplemental figure 5

	Protein accession	Protein name	UB2_GG	UB4_GG	UB6_GG
1	sp|O77059|CRY1_DROME	cry	0.7509428	0.6806738	0.9077939
2	sp|O97069|ASSY_DROME	Ass	-0.5535982	-0.5781328	-0.5193034
3	sp|P04146|COPIA_DROME	GIP	1.033082	0.573145	0.6086989
4	sp|P20828|GAG2_DROME	gag	-1.2239952	-1.0376151	-1.1001391
5	sp|P33269|CP4D1_DROME	Cyp4d1	-0.4571317	-0.6557903	-0.5076349
6	sp|Q02910|CPN_DROME	Cpn	-0.6991523	-0.4940111	-0.4924277
7	sp|Q8I7Q0|GAGO_DROME	gag	0.691283	0.7828238	0.7342404
8	sp|Q9V558|CP4P1_DROME	Cyp4p1	-0.8989049	-1.0312184	-0.9092534
9	sp|Q9V773|C6A20_DROME	Cyp6a20	0.5826323	0.5794641	0.476633
10	sp|Q9VLC0|VPS54_DROME	scat	-0.5984843	-0.4989365	-0.7019897
11	sp|Q9VMS9|C4AC1_DROME	Cyp4ac1	0.792654	0.6834434	0.9084801
12	sp|Q9VP04|ORMDL_DROME	ORMDL	-0.5396289	-0.5239099	-0.5993055
13	tr|A0A0A1EI90|A0A0A1EI90_DROME	Myo81F	-0.6467478	-0.8899753	-0.668474
14	tr|A0A0K0K2E3|A0A0K0K2E3_DROME	ND4	-0.856811	-0.5379381	-0.7090508
15	tr|A1Z6S2|A1Z6S2_DROME	CheB42c	0.4874266	0.4565415	0.6254896
16	tr|C9QP70|C9QP70_DROME	CG43402	0.4933459	0.5374567	0.6040838
17	tr|D5SHN8|D5SHN8_DROME	CG42367-RB	-1.113475	-1.0704593	-1.1946644
18	tr|D7QZ91|D7QZ91_DROME	gag	1.2613679	2.7033938	0.9700819
19	tr|E1JIZ9|E1JIZ9_DROME	trv	-0.7536639	-0.740729	-0.9494164
20	tr|G2J5U6|G2J5U6_DROME	CG15657-RA	-0.6697946	-0.5073143	-0.7996639
21	tr|P92181|P92181_DROME	Lcp65Ac	-1.1662225	-0.4912953	-0.7224925
22	tr|Q3HKQ6|Q3HKQ6_DROME	CG11598	1.8919162	1.6381224	1.8540794
23	tr|Q4LDP4|Q4LDP4_DROME	DS02252	-0.5917436	-0.7017037	-0.4800633
24	tr|Q4V512|Q4V512_DROME	CG14934	-0.7965781	-0.7398368	-0.7927147
25	tr|Q4V5F2|Q4V5F2_DROME	CG15661	-0.8821353	-0.8710654	-0.6306601
26	tr|Q4V5F7|Q4V5F7_DROME	CG13155	2.3375894	2.4668095	2.538089
27	tr|Q59DY1|Q59DY1_DROME	CG14398	-0.576575	-0.6301862	-0.4835909
28	tr|Q5BI62|Q5BI62_DROME	Mdr50	0.6373579	0.5607585	0.5352546
29	tr|Q5BI69|Q5BI69_DROME	GstE5	0.7962674	0.6865229	0.7890578
30	tr|Q7JVX3|Q7JVX3_DROME	BEST	-0.4676864	-0.8157421	-0.6082346
31	tr|Q7JWW6|Q7JWW6_DROME	Ady43A	-0.5372475	-0.601009	-0.4563347
32	tr|Q7KK90|Q7KK90_DROME	GstE1	0.5591688	0.536908	0.7426363
33	tr|Q8IQ80|Q8IQ80_DROME	pst	-0.697647	-0.5098389	-0.7488465
34	tr|Q8SZG4|Q8SZG4_DROME	Jon25Bi	-1.4739568	-1.4120246	-1.0673406
35	tr|Q8T0X2|Q8T0X2_DROME	GstE8	0.766097	0.4586324	0.6850813
36	tr|Q8T3S2|Q8T3S2_DROME	CG5539	0.7783225	0.7849892	0.909824
37	tr|Q8T8R2|Q8T8R2_DROME	GstE8	0.766097	0.4586324	0.6850813
38	tr|Q8T8W0|Q8T8W0_DROME	CG2127	0.7150316	0.6413825	0.7114019
39	tr|Q8T9C4|Q8T9C4_DROME	NA	-0.6756966	-0.6438735	-0.6333041
40	tr|Q9TNT1|Q9TNT1_DROME	NA	0.6508455	0.8356499	0.5965573
41	tr|Q9VGL0|Q9VGL0_DROME	CG14712	-0.6609237	-0.6044155	-0.5726776
42	tr|Q9VIB1|Q9VIB1_DROME	alpha-Est9	-0.6355758	-0.5548752	-0.5710876
43	tr|Q9VJ45|Q9VJ45_DROME	Ugt36E1	2.2527082	2.2994517	2.307663
44	tr|Q9VL67|Q9VL67_DROME	CG4594	-0.6417236	-0.7811471	-0.7577288
45	tr|Q9VLU3|Q9VLU3_DROME	CG12560	1.707294	1.7319477	1.5982661
46	tr|Q9VS41|Q9VS41_DROME	unc-13-4A	-0.4737688	-0.4519142	-0.5221981
47	tr|Q9VVZ4|Q9VVZ4_DROME	CG14095	0.4659586	0.6695614	0.6939991
48	tr|Q9VXV4|Q9VXV4_DROME	CG11655	0.8033358	0.4826198	0.5364396
49	tr|Q9VYS9|Q9VYS9_DROME	CG10352	0.6543305	0.5054921	0.917353

Supplemental figure 5: List of proteins intersecting in figure 2C and their directionality of change.

Supplemental figure 6

Up- and down-regulated proteins in each group

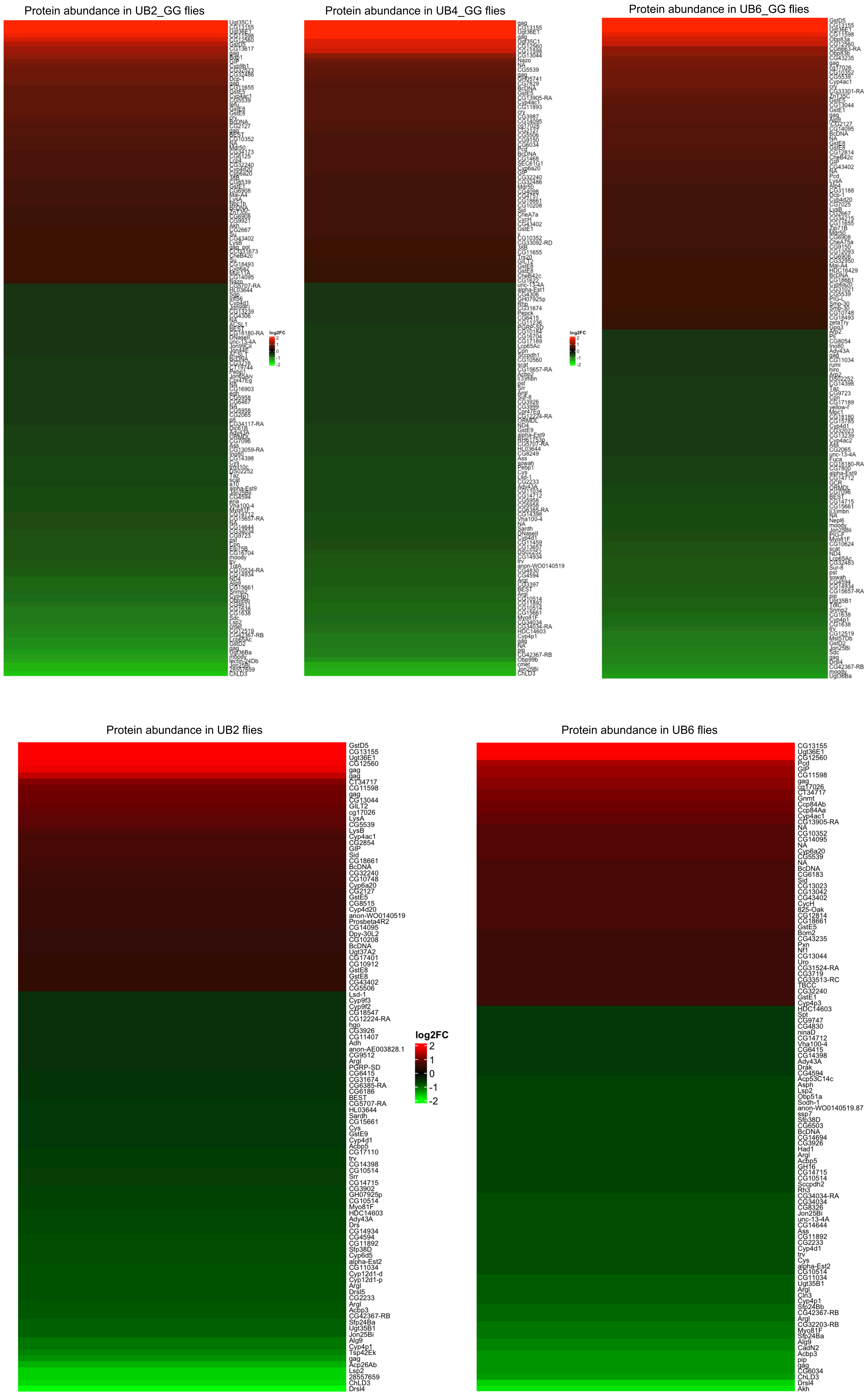

Supplemental figure 6: Heatmaps of all proteins that were up- or down-regulated in the presence of specific UBX linear proteins.

Supplemental figure 7

STRING database outcome of the data from figure 6B.

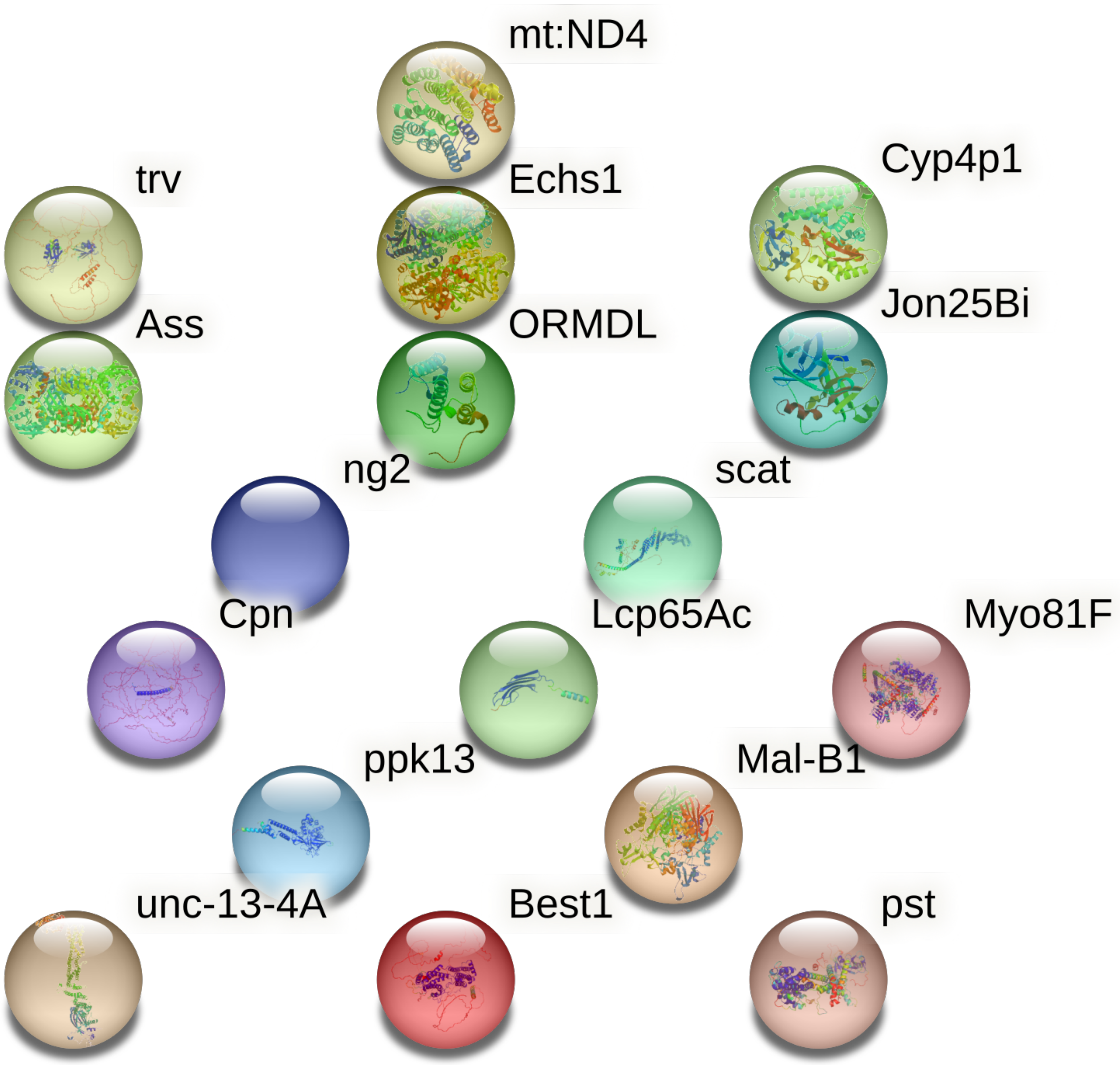

Supplement: jkae209_Supplementary_Data [file Supplemental_Figures_1-7_G3-2024-405312.pdf › Supplemental_Figures_1-7_G3-2024-405312.pdf]
